# Surgical decision-making and referral patterns for adrenal vein sampling in primary aldosteronism: a national survey of endocrine surgeons

**DOI:** 10.3389/fendo.2026.1909164

**Published:** 2026-10-01

**Authors:** Marisela Neff, Konrad Kozlowski, Chloe Issa, Anmol Patel, Jack Lin, Hannah Clode, Thomas Pennix, Zeina Hannoush, Alejandro Ayala, John I. Lew, Zoe A. Miller

**Affiliations:** 1 University of Miami Leonard M. Miller School of Medicine, Miami, FL, United States; 2 Division of Endocrinology, Diabetes and Metabolism, University of Miami Health, Miami, FL, United States; 3 Division of Endocrine Surgery, DeWitt Daughtry Family Department of Surgery, University of Miami Health, Miami, FL, United States; 4 Department of Interventional Radiology, University of Miami Health System/Jackson Memorial Hospital, Miami, FL, United States

**Keywords:** adrenal venous sampling, endocrine surgery, interventional radiology, primary aldosteronism, referral

## Abstract

**Background:**

This study aimed to discover and explore factors associated with endocrine surgeons’ (ES) referral patterns to Interventional Radiologists (IR) for adrenal vein sampling (AVS) for patients with primary aldosteronism (PA).

**Methods:**

Following IRB approval, an electronic survey was distributed to members of the American Association of Endocrine Surgeons (AAES). This cross-sectional, descriptive survey was designed to generate hypotheses about AVS referral behavior rather than test prespecified causal relationships. Data collected included demographics, practice type, referral patterns, opinions on PA management, AVS utilization, confidence levels in AVS and Interventional Radiology (IR) proceduralists, and satisfaction with AVS results.

**Results:**

107 of 806 AAES members responded, 100 (93.5%) of which were ES. Of those surveyed, 45% (n=48) had 10+ years of practice experience, with 61% (n=74) at academic centers. Specialists managed 7.54 (± 5.71) PA patients per year. Among these surgeons, 71% reported “always” ordering AVS for PA, while 28% stated they “sometimes” ordered AVS. If bilateral adrenal nodules were identified on imaging, 100% of surgeons referred patients for AVS, whereas 78% referred patients with unilateral adrenal lesions. Among this group, 56% reported they trusted their IR physician of choice to perform AVS whereas 31% replied they trusted the associated IR group. AAES respondents “always” referred patients to local academic centers (71%), and “never” referred patients to local private IR groups (75%) or distant academic centers (75%). Among respondents, 83% were satisfied with AVS results.

**Conclusions:**

AVS plays a central role in surgical decision-making for primary aldosteronism. Endocrine surgeons (ES) demonstrate strong referral patterns favoring individual interventional radiologists at local academic centers, with referral behavior affected by institutional context, trust in proceduralists and perceptions of procedural standardization. These findings reflect self-reported referral patterns among AAES members and may not capture actual observed clinical behavior.

## 1 Introduction

Primary aldosteronism (PA) is a common cause of secondary hypertension, estimated to affect approximately 6% of patients with essential hypertension and up to 20% of patients with resistant hypertension (1). Over 90% of PA cases are caused by overproduction of aldosterone by one or both adrenal glands. Differentiating between unilateral and bilateral disease is integral to management as patients with a unilateral aldosterone producing adenoma (APA) are candidates for adrenalectomy while treatment of bilateral primary aldosteronism consists of optimization of medications (2, 3). In patients with unilateral APA’s, Adrenalectomy has a 95% chance at a complete biochemical “cure” (normalization of hypokalemia and aldosterone-to-renin serum ratio) and a 40% chance at clinical cure or improvement (normalization of blood pressure with decrease in or cessation of antihypertensive medications) (4–7).

While CT and MRI images can demonstrate the presence of an adrenal adenoma, discordance between imaging and true lateralization of disease is not uncommon (8). Adrenal Vein Sampling with its high sensitivity and specificity, has become the gold standard for lateralization of disease (9, 10). Despite guidelines recommending AVS, aspects of the procedure remain controversial, which may affect its true use in practice. AVS is technically demanding with average success rates that fluctuate greatly depending on the experience of the Interventional Radiologist performing the procedure. AVS technique, protocols used and interpretation criteria of results differ greatly by proceduralist and institution (11–14). Usually, the procedure is performed at a tertiary center, access to which may be limited in some rural areas. While minimally invasive with a very high safety profile, AVS is still a procedure and there is some inherent risk to the patient (15). These perceived limitations may decrease the utility and referrals to AVS to guide in the treatment of PA.

While safety, efficacy and procedural outcomes have been extensively studied, little is known about how referring surgeons make decisions regarding AVS utilization. We aimed to characterize endocrine surgeons’ AVS referral patterns and to explore whether factors such as IR practice setting, location and ES experience with interventional radiologists are associated with referral behavior for patients with primary aldosteronism. This was a cross-sectional, descriptive survey designed to generate hypotheses about AVS referral behavior. Factors influencing referral behavior are examined, including opinions on the procedure itself, proceduralist groups, practices and institutional categories. Preliminary findings from this study were presented at the Society of Interventional Radiology Annual Scientific Meeting (2024) (16).

## 2 Materials and methods

This study was approved by the University of Miami Institutional Review Board. It involved an anonymous, voluntary electronic survey of AAES members conducted via the Qualtrics platform. No incentives were offered for answering the survey. No participant-identifying data (names, email addresses or IP addresses) and no patient-level data were shared. Completion of the survey implied consent; the IRB granted a waiver of written informed consent due to minimal risk and the impracticability of obtaining signed consent from all AAES members. Approval was obtained from the AAES Research Committee. The study was conducted in accordance with the World Medical Association’s Declaration of Helsinki.

### 2.1 Survey development and pre-testing

Survey questions were drafted jointly by specialists in Interventional Radiology, Endocrine Surgery and Endocrinology and iteratively refined. A pilot was performed with distribution of the second version of the survey. Two Endocrinologists and two Endocrine Surgeons participated in the pilot, the analysis of which resulted in two more iterations with version 4 of the survey distributed to the AAES. The functionality of the survey was tested by three authors of the study who confirmed ability to go through and answer all questions without technical failure. The full survey instrument is provided in **Supplementary Material 1** (**Supplementary Material 1**: AVS Questionnaire).

### 2.2 Structure

The final survey contained 44 questions which were non-randomized and adaptive questioning was utilized. Several items allowed respondents to select more than one answer choice (e.g., reasons for ordering AVS, perceived barriers, desired improvements); for these questions, percentages may sum to more than 100%. When completing the survey, additional questions were administered based on prior responses. Therefore, the minimal number of questions asked on a completed survey was 14. Participants were able to review and change their answers via a “Back” button and move ahead without answering a question by pressing a “Next” button. The number of questions displayed per page varied whether the study was answered on a mobile device versus a tablet or laptop computer.

### 2.3 Content

Questions collected data regarding AAES member demographics, practice area, referral patterns, opinions about PA management, utilization of AVS, confidence levels in AVS and IR proceduralists, and satisfaction with AVS results. Practice location and training were categorized by US census region (West, Midwest, Southeast, etc.).

### 2.4 Survey participants, recruitment and administration

The survey was distributed via email to all 806 members of the American Association of Endocrine Surgeons (AAES), which included endocrine surgeons, general surgeons, as well as surgical oncologists, among others. The survey was also distributed to AAES members via the social media platform X, formerly known as Twitter. The email to all participants explained the survey rationale, credentials and contact details of the principal investigator, and contained a unique email-specific questionnaire survey link which allowed for web-based response to the survey. Email invitations contained unique Qualtrics links that allowed only one submission per invitee. The social media distribution used a generic link; Qualtrics settings restricted multiple submissions from the same IP address, but duplicates across devices or networks could not be fully excluded. The survey was initially emailed and announced on social media on August 2, 2023. The survey was conducted through September 5, 2023, with a single reminder email that was sent out 3 weeks after the initial date.

### 2.5 Data handling and analysis

Survey links were not utilized to record email or IP address from which the survey was accessed. Cookies were used to assign a unique identifier to each client computer.

Responses to the questionnaire were automatically captured and entered into Qualtrics. Within Qualtrics, this data could be manipulated into tables. Utilizing Qualtrics analytic tools, obvious duplicate patterns were checked for but none were found.

### 2.6 Statistical analysis

Demographics of survey responders as well as other survey questions posed as “yes/no” or involving multiple answer choices were presented as percentages for categorical variables. Continuous variables were reported as mean and standard deviation. All data analyses were performed using SPSS Version 26 (IBM Corp., Armonk, N.Y., USA).

## 3 Results

The survey was sent to 806 AAES members, of whom 107 completed at least one survey section (overall response rate 13.3%). Because incomplete questionnaires were included, denominators vary by item; the number of respondents for each question is reported in the text, tables, or figure captions. Participants included Endocrine Surgeons (n=100, 93%), General Surgeons (n=4, 4%), and other (n=3, 3%) specialists including one urologist and one surgical oncologist (**Table 1**). Incomplete questionnaires were included in analysis. Practice experience varied from 0–5 years (n=43, 40.2%), 6–10 years (n=16, 15%), 11–15 years (n=18, 16.8%), and >15 years (n=30, 28%). Most members practiced at academic/university hospitals (n=74, 61.6%), or community hospitals (n=25, 20.7%) with the least practicing in the private sector (n=15, 12.4%). Survey respondents practiced all over the United States, most commonly the Midwest (n=26, 25.74%), Southeast (n=22, 21.78%), and Southwest/Mid-Atlantic (n=14, 13.86%). The most common practice size was 3–5 practitioners (n=45, 42.86%), followed by 5–10 practitioners (n=20, 19.1%), >10 practitioners (n=14, 13.3%), individual practitioner (n=14, 13.3%), and 2 practitioners (n=12, 11.4%).

**Table 1:** Demographics of referring surgeons.

Sex n (%)	Count (%)
Female	47 (44%)
Male	57 (53%)
Specialty n (%)	
Endocrine Surgery	100 (93%)
General Surgery	4 (4%)
Other	3 (3%)
Duration of practice n (%)	
0–5 years	43 (40%)
6–10 years	16 (15%)
11–15 years	18 (17%)
>15 years	30 (28%)
Practice type n (%)	
Community Hospital	25 (21%)
Academic/University Hos	74 (62%)
Private Practice	15 (12%)
Other	7 (6%)
Practice size n (%)	
Individual	14 (13%)
2 practitioners	12 (11%)
3–5 practitioners	45 (43%)
5–10 practitioners	20 (19%)
10+ practitioners	14 (13%)
Practice area n (%)	
West	11 (11%)
Northwest	6 (6%)
Southwest	14 (14%)
Midwest	26 (26%)
Southeast	22 (22%)
Mid Atlantic	14 (14%)
North East	8 (18%)

AAES members treated a mean of 7.54 (± 5.71) patients with PA per year. Among respondents who answered this item, 93/101 (92.1%) ordered imaging during the pre-operative evaluation, preferring CT 88/100 (88%) over MRI (**Table 2**). Among respondents who answered the AVS frequency item, 70/98 (71.4%) reported ‘always’ ordering AVS, while 27/98 (27.6%) reported ‘sometimes’ ordering AVS. Among respondents who answered these management items, 99/99 (100%) selected AVS for bilateral nodules, whereas 77/99 (77.8%) selected referral for AVS for unilateral nodules (**Figure 1**). Only 1/99 (1.2%) of surgeons performed unilateral adrenalectomy in patients with a unilateral nodule identified on imaging without prior AVS. When asked why they sent patients with PA for AVS, among respondents who answered this multi-select item, 88/98 (90%) selected “I think the procedure appropriately differentiates patients who would benefit from surgery vs medical treatment and has a significant effect on management. Among respondents who answered this multi-select item, 60/98 (61.2%) selected “believe the results of AVS are accurate,” 47/98 (48.0%) selected “believe the results of AVS are reproducible,” and 61/98 (62.2%) selected they were “pleased with the procedure success rates” they experienced. While 56/98 (57.1%) “trust the interventional radiologist they send all AVS patients to,” only 31/98 (31.6%) trust the interventional radiology group for their patients needing AVS. Notably, only 33/98 (33.7%) were “pleased with the results after AVS despite the variation in procedure steps and value interpretation” while 49/98 (50%) mentioned “reporting of AVS results from the interventional radiologist(s) made integrating AVS into their management algorithm easy.” (**Figure 2**).

**Table 2:** AVS patient work-up.

Patients treated per year Mean (± SD)	7.54 (± 5.71)
Imaging ordered with workup	
Yes	93 (92%)
No	1 (1%)
Sometimes	7 (7%)
Imaging type	
CT	88 (88%)
MRI	2 (2%)
Either	10 (10%)

**Figure 1:**
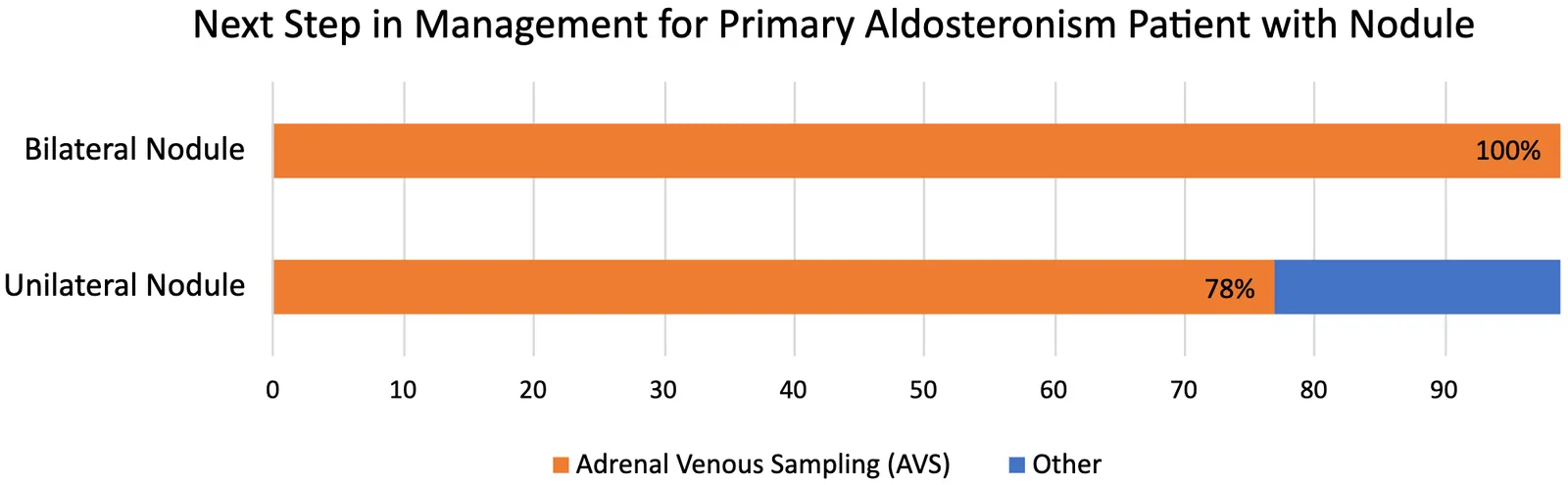
Referring physician management of unilateral vs. bilateral nodules. Total 99 respondents answered this question. Percentages are represented.

**Figure 2:**
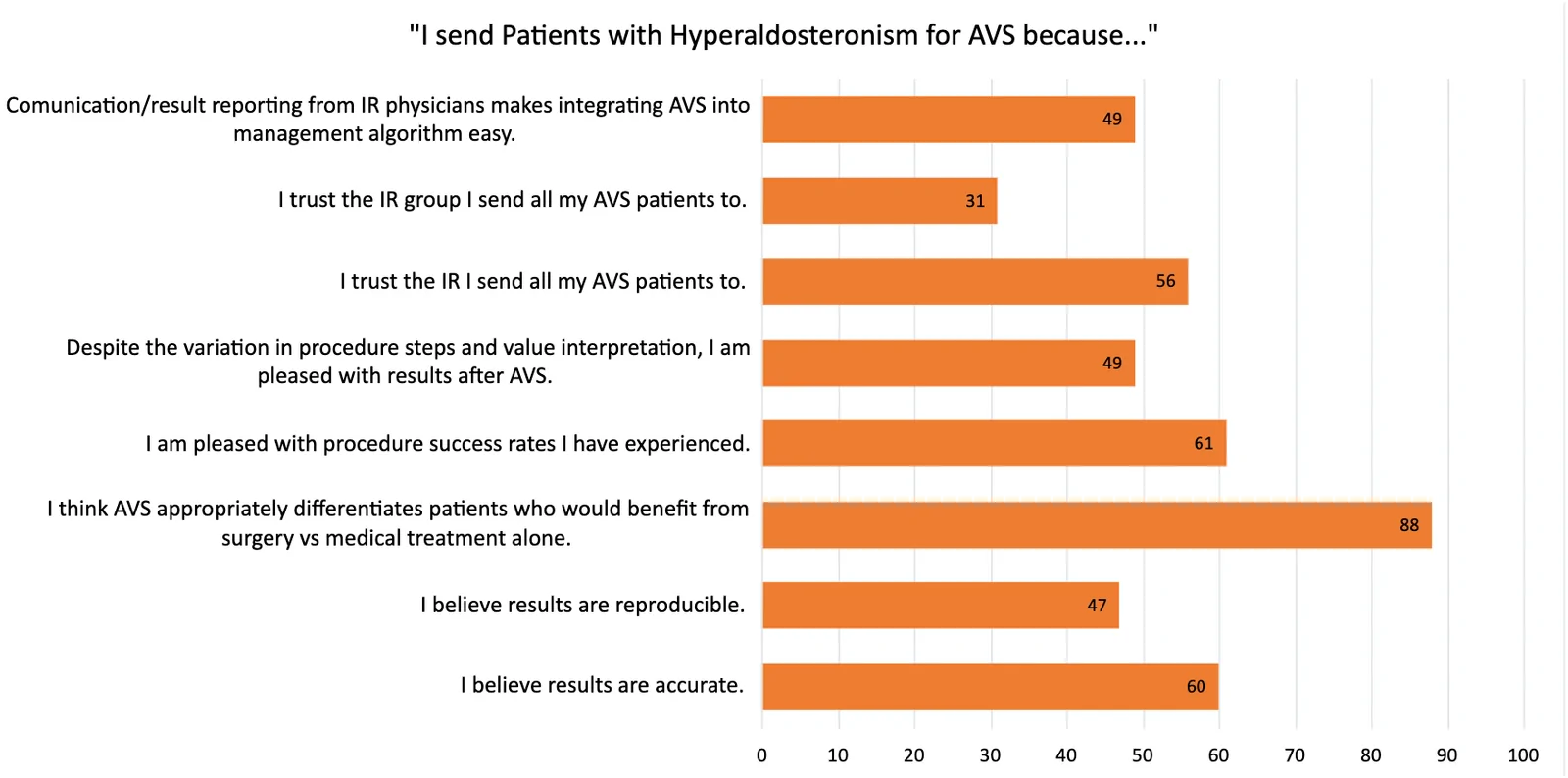
Physician's impressions of AVS procedure value and motivating factors for referral. Among respondents who answered this item; multiple responses were allowed, percentages >100%. Counts are represented.

Of study respondents, 52% strictly followed the Endocrine Society guidelines that “Younger patients (<age 35 years) with spontaneous hypokalemia, marked aldosterone excess, and unilateral adrenal lesions with radiological features consistent with a cortical adenoma on adrenal CT scan may not need AVS before proceeding to unilateral adrenalectomy,” while 48% sometimes took that guideline into account (17).

When referring patients for AVS, surgeons specified that they almost “always” sent them to interventional radiologists at a local academic institution 64/90 (71.1%) and typically “never” sent them to local private interventional radiologists 53/71 (74.7%), distant academic institutions 53/71 (74.7%), or specialized institutions 62/71 (89.9%) (**Figure 3**). Among respondents who answered the satisfaction item, 80/96 (83.3%) were satisfied with AVS results when performed, and 96/96 (100%) felt AVS was helpful in management of patients with PA (**Figure 4**).

**Figure 3:**
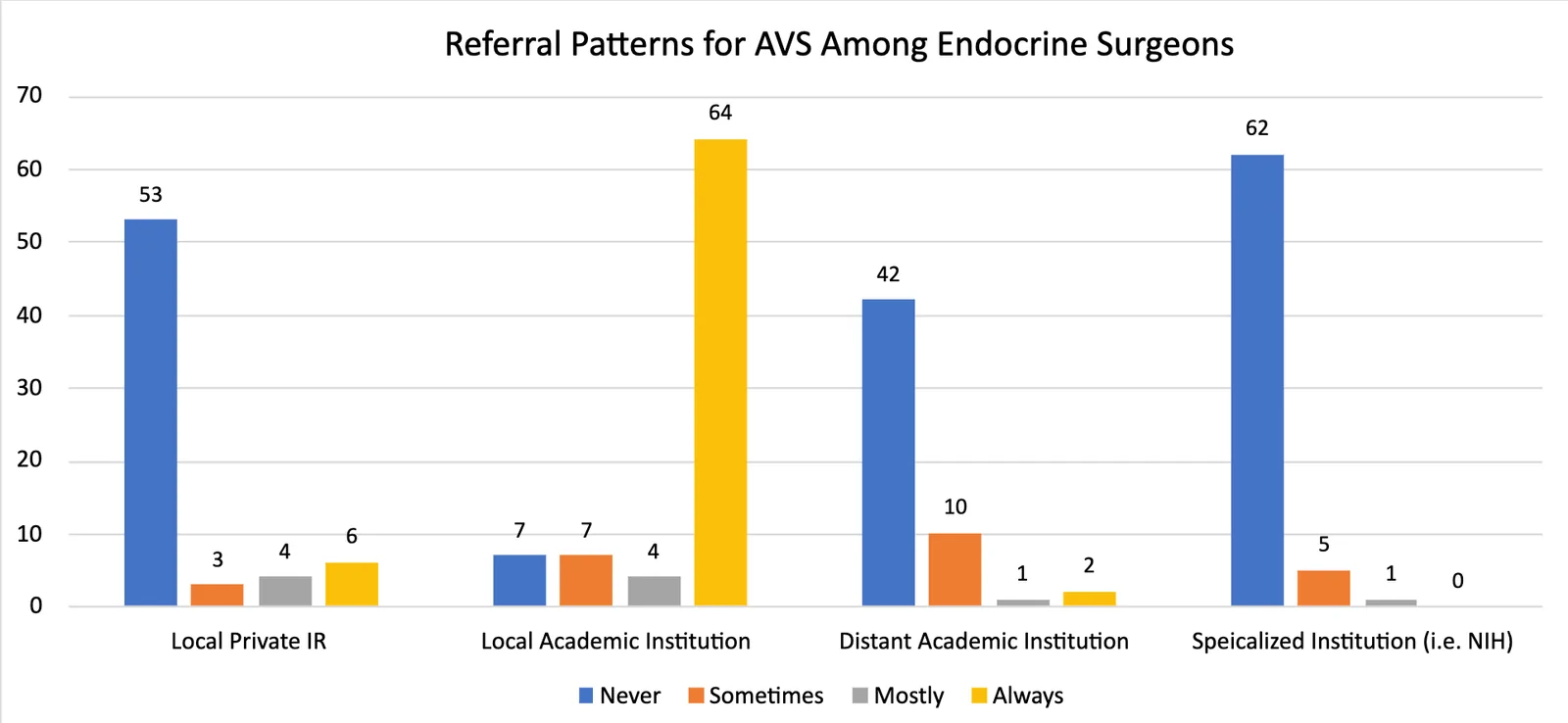
Where do endocrine surgeons refer patients for AVS. Among respondents who answered this item; multiple responses were allowed, percentages >100%. Counts are represented.

**Figure 4:**
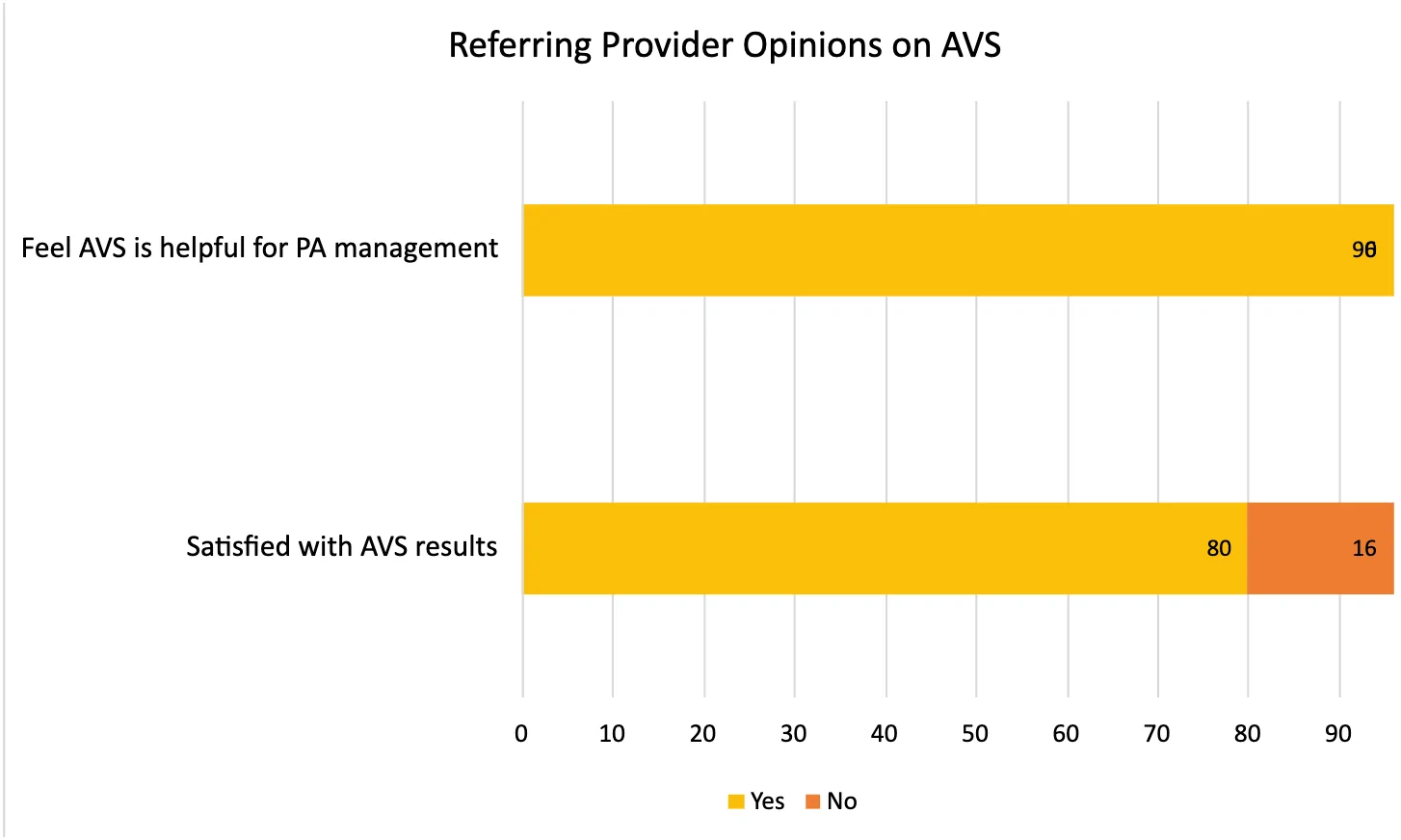
Referring provider AVS satisfaction. Total 96 respondents answered this question. Counts are represented.

As shown in **Figure 5**, when asked about future improvements to be made in AVS in an optional comments section, the most frequent comments centered around formulating a standardized technique/protocol across institutions (n=9, 34.3%), improving organization/labeling of samples for easier interpretation (n=5, 14.3%), and developing an AVS protocol for patients with concomitant hypercortisolism (n=5, 14.3%).

**Figure 5:**
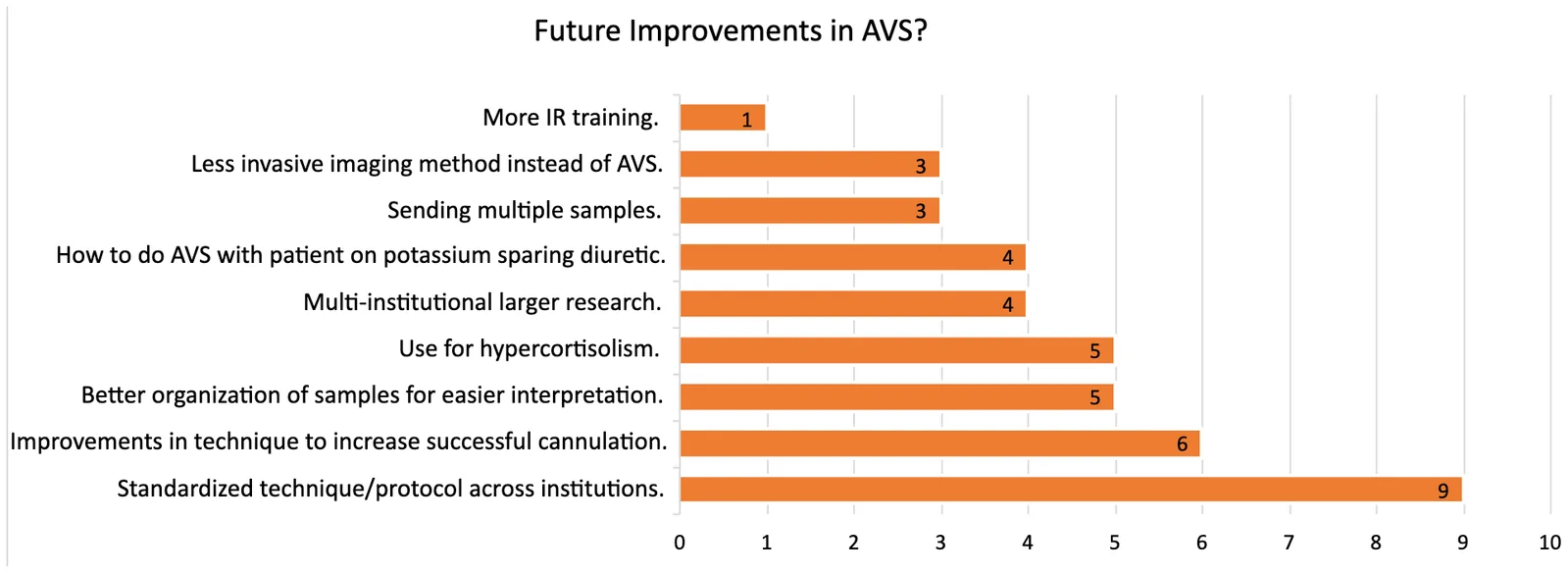
Referring physician impressions regarding future improvements in AVS. Total 59 respondents answered this item in an optional comments section. Counts of summarized responses are represented.

## 4 Discussion

Adrenal vein sampling (AVS) remains the gold standard for lateralization in primary aldosteronism despite limitations such as variability in technique, interpretation, and operator experience. This study focuses on AVS referral behavior among endocrine surgeons, despite the above factors, while discovering additional factors that may prove more influential.

In this cohort, endocrine surgeons reported frequent use of AVS despite known challenges. Respondents consistently viewed AVS as an important tool for distinguishing patients who would benefit from surgical versus medical management, reinforcing its central role in clinical decision-making.

### 4.1 Factors affecting AVS referral behavior

Several factors appear to shape AVS referral patterns. Prior studies have shown AVS success rates correlate with operator and center volume and high-volume academic centers often achieve better cannulation and diagnostic reliability (9, 12, 15). In our survey, Endocrine surgeons report frequent use of AVS, with most respondents referring to individual interventional radiologists at local academic centers and rarely to private or distant centers. This pattern may reflect perceived advantages of and trust in higher-volume operators and multidisciplinary infrastructure. Surgeons more often reported confidence in specific proceduralists than in interventional radiology groups, suggesting that inter-specialty relationships and perceived expertise, rather than institutional affiliation alone, are associated with referral decision.

Institutional context also plays a role. The strong preference for referral to local academic centers likely reflects a combination of accessibility, perceived expertise, and the presence of pre-existing multidisciplinary infrastructure.

Perceptions of procedural standardization do influence how AVS is incorporated into management. While many surgeons reported satisfaction with procedural success rates, confidence decreased when variability in technique and interpretation was taken into account.

### 4.2 Practical implications

These findings suggest several areas for improvement. Although overall satisfaction with AVS was high, this dropped when variability in technique and interpretation was considered, highlighting the need for greater standardization. When given the chance to leave open comments, respondents emphasized how helpful it is to receive clear communication of results from interventional radiologists. They also chose to comment on the importance of continued teaching of AVS technique to interventional radiology trainees.

### 4.3 Additional considerations

Some respondents also mentioned the utility of AVS in determining gland dominance in patients with hypercortisolism. While there is evidence supporting the role of AVS in some cases, protocols and interpretation remain less standardized than in primary aldosteronism. Patients with co-secretion of aldosterone and cortisol present additional complexity, and further study in these areas is warranted.

### 4.4 Limitations

This study has several limitations. The overall response rate was relatively low (13.3%), and the survey was restricted to members of a single subspecialty organization. This may limit generalizability and introduce selection bias toward surgeons who are more engaged in adrenal disease management. Although email invitations used unique links and Qualtrics settings restricted multiple submissions from the same IP address, duplicate responses across devices or networks cannot be completely excluded due to the distribution of the generic social media link. We did not collect data on individual interventional radiology operator or center AVS volume or reported cannulation success rates. These have been associated with AVS quality in prior studies and represent a potential unmeasured factor influencing referral patterns. Because AVS use and referral behavior were self-reported, recall bias and discrepancies between stated and actual practice were not possible. We were also unable to confirm AVS outcomes or whether reported referrals resulted in completed procedures. The survey instrument underwent content review and informal piloting but was not formally validated, and we did not limit participation to one surgeon per institution, raising the possibility of institutional clustering. Despite these limitations, respondents represented a geographically diverse group of US clinicians actively managing PA, and the survey provides a descriptive snapshot of current practice patterns at the time of data collection.

### 4.5 Broader implications

While this study does not introduce new procedural techniques or diagnostic criteria for AVS, it provides insight into the real-world decision-making processes that shape its use. The findings suggest that variability in AVS utilization may be affected less by disagreement with guidelines and more by differences in multidisciplinary trust structures and institutional organization.

Beyond AVS specifically, these findings highlight how referral decisions for technically complex procedures are influenced by interpersonal trust, institutional infrastructure, and perceived standardization. These factors likely extend beyond primary aldosteronism and may influence adoption of a variety of procedures across multiple multidisciplinary clinical settings. Prior literature similarly suggests that referral decisions are shaped by perceived expertise, interpersonal relationships, communication, and system affiliation (18–20).

In conclusion, AVS remains a critical component of surgical decision-making in PA. Referral behavior appears to be associated with trust in individual proceduralists, local institutional context, and perceptions of procedural standardization. Addressing these factors may help improve collaboration, streamline referral pathways, and expand access to AVS. Future work should examine how metrics such as operator AVS volume or cannulation success rates may shape referral patterns or act as bottlenecks in access to care.

## Data Availability

The raw data supporting the conclusions of this article will be made available by the authors, without undue reservation.
