# Supplementary material for: Surgical decision-making and referral patterns for adrenal vein sampling in primary aldosteronism: a national survey of endocrine surgeons
Source: Front Endocrinol (Lausanne). 2026 Oct 1;17:1909164. doi: 10.3389/fendo.2026.1909164 (PMC13670166; doi:10.3389/fendo.2026.1909164)
Supplement: Supplementary file 1 [file DataSheet1.pdf]

AVS Questionnaire

Start of Block:

Q1 What is your sex?

- ○ Male
- ○ Female
- ○ Prefer not to answer
- ○ Not Listed ____

Q2 What is your specialty?

- ○ Endocrine Surgery
- ○ General Surgery
- ○ Urology
- ○ Other ____

Q3 How many years have you been in practice?

- ○ < 1 y
- ○ 1-5 y
- ○ 6-10 y
- ○ 11-15 y
- ○ > 15 y

Q4 What is your current practice type? (select all that apply)

- ☐ Community Hospital
- ☐ Academic / University Hospital
- ☐ Group Private Practice
- ☐ Individual Private Practice
- ☐ Other

Q5 Please use this map when answering the next two questions.

Q6 Please select in which area(s) you completed your training:

- ☐ North West
- ☐ West
- ☐ South West
- ☐ Midwest
- ☐ Southeast
- ☐ Mid-Atlantic
- ☐ North East

Q7 Please select the area you currently practice:

- North West
- West
- South West
- Midwest
- Southeast
- Mid-Atlantic
- North East

Q8 What is your current practice size?

- Individual
- 2 Practitioners
- 3-5 Practitioners
- 5-10 Practitioners
- > 10 Practitioners

*

Q9 **Approximately how many patients with Primary Hyperaldosteronism have you treated in the past year?**

____

Q10 **Do you order imaging during the patient workup**?

- ◯ Yes
- ◯ Sometimes
- ◯ No
- ◯ No- patient arrives with imaging already performed

Display This Question:

If Do you order imaging during the patient workup? = Yes

Q11 **If you order imaging, which modality do you prefer? (Select all that apply)**

- ◯ CT
- ◯ MRI
- ◯ Either CT or MRI (considered equivalent in your evaluation)
- ◯ Other: Please Specify ____

Display This Question:

If Do you order imaging during the patient workup? = Yes

Q13 **If a unilateral nodule is found, what is your next step in management?**

- ◯ refer for Adrenal Venous Sampling
- ◯ perform unilateral adrenalectomy
- ◯ Pharmacotherapy
- ◯ plasma aldosterone/renin ratio (ARR) testing
- ◯ Other ____

Display This Question:

If Do you order imaging during the patient workup? = Yes

Q14 **If bilateral nodules are found, what is your next step in management?**

- ○ Adrenal Venous Sampling
- ○ Pharmacotherapy
- ○ Other ____

Display This Question:

If Do you order imaging during the patient workup? = No

Q16 **If no, please select all reasons that apply.**

- ▢ Do not feel imaging is necessary for diagnosis
- ▢ Do not feel imaging is necessary for treatment
- ▢ Other

Display This Question:

If If no, please select all reasons that apply. = Other

Q17 **If other, please specify:**

____

Page Break

Q18a **Do you routinely order Adrenal Venous Sampling (AVS) as part of the management for Hyperaldosteronism?**

- ◯ Yes
- ◯ No

Display This Question:

If Do you routinely order Adrenal Venous Sampling (AVS) as part of the diagnosis and management for... = Yes

Q18b **Please select all that apply-**
I send patients with Hyperaldosteronism for Adrenal Vein Sampling because:

- ☐ I find that the procedure appropriately differentiates patients who would benefit from surgery from those who would benefit from medical treatment alone, and therefore has a significant effect on proper management
- ☐ I believe the results are accurate
- ☐ I believe the results are reproducible
- ☐ I am pleased with the procedure success rates I have experienced
- ☐ Despite the variation in procedure steps and value interpretation, I am pleased with the results after Adrenal Vein Sampling
- ☐ I trust the interventional radiologist I send all my adrenal vein sampling patients to
- ☐ I trust the interventional radiology group I send all my adrenal vein sampling patients to
- ☐ Communication / result reporting from the physicians I refer my patients to, makes integrating Adrenal Vein Sampling into my management algorithm easy
- ☐ Click to write Choice 9

Display This Question:

If Do you routinely order Adrenal Venous Sampling (AVS) as part of the diagnosis and management for... = Yes

Q19 **When referring patients for Adrenal Vein Sampling, what type of practitioner or institution do you refer them to?**

	Never	Sometimes	Mostly	Always
Local private Interventional Radiologist	o	o	o	o
Local Academic Institution / University Interventional Radiologist	o	o	o	o
Distant Academic Institution / University Interventional Radiologist	o	o	o	o
Specialized Institution (i.e. NIH)	o	o	o	o
Other	o	o	o	o

Page Break

Display This Question:

If When referring patients for Adrenal Vein Sampling, what type of practitioner or institution do yo... = Local private Interventional Radiologist

Q20 **Do you send patients to the same practitioner(s) for Adrenal Vein Sampling?**

- ○ Same single Interventional Radiologist
- ○ Same Interventional Radiology Group (Private or Academic)
- ○ Different practices

Display This Question:

If Do you routinely order Adrenal Venous Sampling (AVS) as part of the diagnosis and management for... = No

Q21 **If no, please select all reasons that apply:**

- ☐ I doubt the accuracy of the procedure
- ☐ I doubt the reproducibility of the procedure
- ☐ The rate of success in obtaining blood from both adrenal veins is too low to warrant referral
- ☐ Lack of standardization of the steps of the procedure limits accuracy and/or reproducibility of results
- ☐ Lack of standardized cutoff values for selectivity and lateralization indexes limits utility of procedure
- ☐ Communication, including result reporting, from the interventional radiologist is subpar
- ☐ No known interventionist / center to refer to that is within easy traveling distance from my practice
- ☐ The results do not significantly affect management
- ☐ I am rarely satisfied with the results of Adrenal Vein Sampling, whether successful or not
- ☐ Not available at my institution
- ☐ Not cost effective
- ☐ Unsafe procedure
- ☐ Other

Display This Question:

If If no, please select all reasons that apply: = Other

Q22 **If other, please specify:**

____

Page Break

Display This Question:

If Do you routinely order Adrenal Venous Sampling (AVS) as part of the diagnosis and management for... = No

Or When referring patients for Adrenal Vein Sampling, what type of practitioner or institution do yo... = Specialized Institution (i.e. NIH)

Q23 **If there was an Interventional Radiologist near you who performed Adrenal Vein Sampling, what rate of success in sampling both the right and left adrenal veins would you accept as sufficient to send patients to them?**

At or above:

- ◯ 95% sampling success
- ◯ 90% sampling success
- ◯ 85% sampling success
- ◯ 80% sampling success
- ◯ 75% sampling success
- ◯ 70% sampling success
- ◯ None of the above

Display This Question:

If If there was an Interventional Radiologist near you who performed Adrenal Vein Sampling, what rat... = None of the above

Q24 **If none, please select all reasons that apply.**

☐ 95% success rate is not high enough

☐ Will only refer to specialty research center

☐ No desire to partner with unknown physician

☐ Other

Display This Question:

If If none, please select all reasons that apply. = Other

Q25 If other, please specify:

____

Display This Question:

If If none, please select all reasons that apply. = 95% success rate is not high enough

Q26 **What AVS percent success rate would warrant utility of AVS for you and your practice?**

____

Q27 **Are you satisfied with the results when AVS is performed?**

○ Yes

○ No

Q28 **Do you feel AVS is helpful in management of patients with hyperaldosteronism?**

○ Yes

○ No

Q34 What improvements / research information would you like to see in Adrenal Vein Sampling?

____

Q35 Additional thoughts or comments on Adrenal Vein Sampling and the future?

____

End of Block:
